# Bacterial profiles and their antibiotic susceptibility patterns in neonatal sepsis at the University of Gondar Comprehensive Specialized Hospital, Ethiopia

**DOI:** 10.3389/fmicb.2024.1461689

**Published:** 2024-10-21

**Authors:** Teshiwal Deress, Gizeaddis Belay, Getahun Ayenew, Worku Ferede, Minichile Worku, Tigist Feleke, Meseret Mulu, Solomon Belay, Michael Getie

**Affiliations:** ^1^Department of Quality Assurance and Laboratory Management, School of Biomedical and Laboratory Science, College of Medicine and Health Sciences, University of Gondar, Gondar, Ethiopia; ^2^Department of Medical Microbiology, Amhara National Regional State Public Health Institute, Bahir Dar, Ethiopia; ^3^Department of Molecular Laboratory, Trachoma Elimination Program, The Carter Center, Bahir Dar, Ethiopia; ^4^Microbiology Laboratory, University of Gondar Comprehensive Specialized Hospital, Gondar, Ethiopia

**Keywords:** neonate sepsis, bacterial profiles, antibiotic susceptibility, University of Gondar Comprehensive Specialized Hospital, Ethiopia

## Abstract

**Background:**

Neonatal sepsis is a major cause of morbidity and mortality worldwide. Understanding the bacterial profiles and antibiotic susceptibility patterns causing neonatal sepsis is crucial for guiding appropriate treatment, improving patient outcomes, and combating the emergence of antibiotic resistance. Despite its importance, data regarding neonatal sepsis in the study area is limited. Therefore, this study aimed to characterize the bacterial pathogens and identify associated factors among neonates with suspected sepsis at the University of Gondar Comprehensive Specialized Hospital, Ethiopia.

**Methods:**

A cross-sectional study was conducted by reviewing laboratory records of neonates admitted for suspected sepsis from January 2019 to December 2021. Data were checked for completeness and encoded in a spreadsheet program. Then, data were exported to STATA version 17 for analysis. Descriptive statistics such as frequency and percentage were computed. The association between neonatal sepsis and potential risk factors was assessed using Pearson’s chi-square test. A *p*-value of < 0.05, was considered statistically significant.

**Results:**

A total of 1,236 neonates were included. Of these, 96.2% (1,190/1,236) had a fever before admission. The prevalence of culture-confirmed sepsis was 25.4% (314/1,236). Bacterial pathogens accounted for 23% (284/1,236) of these isolates, with Gram-negative bacteria being more prevalent at 75.3% (214/284) than Gram-positive bacteria at 24.7% (70/284). The most frequently isolated bacterial pathogens were *K. pneumoniae* 38.7% (110/284) and *S. aureus* 13% (37/284). The isolates demonstrated a high resistance level to commonly used antibiotics, with 61.6% exhibiting multidrug resistance. *K. pneumoniae* showed the highest rate of multidrug resistance (90.9%). Neonatal sepsis was associated with several factors, including fever before and after admission, hypothermia, increased respiration, suspected pneumonia, and suspected meningitis.

**Conclusion:**

This study identified a high prevalence of culture-confirmed sepsis in neonates at UoGCSH, with Gram-negative bacteria, especially *K. pneumoniae*, dominating the isolated pathogens. The isolated bacteria exhibited alarming resistance to commonly used antibiotics, with a high proportion demonstrating multidrug resistance. Implementing effective antibiotic stewardship programs is crucial to optimize antibiotic use, reduce unnecessary prescriptions, and curb the spread of resistant strains.

## Background

Neonatal sepsis is a significant global health challenge, particularly in developing countries, where it is the leading cause of morbidity and mortality. It accounts for an estimated 3 million cases annually with a high mortality rate of 11–19% (Glaser et al., 2021; Fleischmann-Struzek et al., 2018). Immature immune systems and barriers heighten neonatal susceptibility to infections (Georges Pius et al., 2022). Sub-Saharan Africa bears a substantial burden, with neonatal sepsis leading to an estimated annual loss of 5.3–8.7 million disability-adjusted life years and an economic impact ranging from $10 billion to $469 billion (Ranjeva et al., 2018; Molloy et al., 2020). This issue is especially pronounced in East Africa, where prevalence reaches 29.7% (Abate et al., 2020).

In Ethiopia, the prevalence of neonatal sepsis is alarmingly high, with rates ranging from 45.8 to 78.3% in different regions (Bayih et al., 2021; Belachew and Tewabe, 2020; Mustefa et al., 2020; Bekele et al., 2022; Roble et al., 2022). This burden is further exacerbated by the increasing antimicrobial resistance (Chaurasia et al., 2019). Multiple risk factors are associated with neonatal infection, including low birth weight (Bayih et al., 2021; Belachew and Tewabe, 2020; Mustefa et al., 2020; Li et al., 2023), maternal history of urinary tract infection (Abate et al., 2020; Bayih et al., 2021; Belachew and Tewabe, 2020; Mustefa et al., 2020), formula feeding, cesarean section (Li et al., 2023), preterm birth (Abate et al., 2020; Bayih et al., 2021; Belachew and Tewabe, 2020; Mustefa et al., 2020; Li et al., 2023; Satar and Özlü, 2012), home delivery, prolonged labor, and premature rupture of membranes (Abate et al., 2020), antenatal urinary tract infection, and intrapartum fever (Bayih et al., 2021; Belachew and Tewabe, 2020; Mustefa et al., 2020). Despite advancements in neonatal care, diagnosing neonatal sepsis remains challenging, underscoring the importance of prompt antibiotic treatment (Ershad et al., 2019).

Regional variations exist in the spectrum of bacterial pathogens causing neonatal sepsis (Poyekar, 2022; Moni et al., 2020). While the predominant bacteria vary geographically, Gram-negative bacteria like *Klebsiella* spp. (Pokhrel et al., 2018; Acheampong et al., 2022; Bhat et al., 2011; Almohammady et al., 2020; Fenta et al., 2022; Pataskar et al., 2023; Bai et al., 2021; Siddiqui et al., 2023; Weldu et al., 2020), *E. coli* (Pokhrel et al., 2018; Acheampong et al., 2022; Bhat et al., 2011; Almohammady et al., 2020; Fenta et al., 2022; Pataskar et al., 2023; Bai et al., 2021), and *E. cloacae* complex (Pataskar et al., 2023; Bai et al., 2021) are commonly isolated. Particularly in developing countries, Gram-negative bacteria stand as the leading cause of morbidity and mortality (Kamalakannan, 2018; Bethou and Bhat, 2022). Among Gram-positive isolates, *S. aureus* (Pokhrel et al., 2018; Acheampong et al., 2022; Almohammady et al., 2020; Tessema et al., 2021; Yadav et al., 2018; Negussie et al., 2015) and CoNS (Pokhrel et al., 2018; Acheampong et al., 2022; Almohammady et al., 2020; Tessema et al., 2021; Yadav et al., 2018; Negussie et al., 2015; Aku et al., 2018) are frequently identified. Antibiotic resistance is a significant concern (Negussie et al., 2015), with alarmingly high levels of multidrug-resistant strains negatively impacting treatment outcomes (Uwe et al., 2022). This emphasizes the need for strict antibiotic use guidelines. Isolated bacteria often show high resistance to conventional antibiotics like ampicillin, cephalosporins, and piperacillin-tazobactam (Uwe et al., 2022; Verma et al., 2015). Studies from India report high rates of multidrug resistance in *Acinetobacter* spp. (82%), *Klebsiella* spp. (54%), and *E. coli* (38%) isolates (Chaurasia et al., 2016). Another Indian study found bacterial isolates resistant to aminoglycosides (74%), third/fourth-generation cephalosporins (95%), and carbapenems (56%) (Shah et al., 2022). Others indicate that 54% of isolated bacteria were resistant to at least one antibiotic (Sands et al., 2021). In Iran, *K. pneumoniae* showed the highest resistance to Cefixime (80.6%), while *E. coli* exhibited significant resistance to Ampicillin (61.8%) (Moftian et al., 2023). Even Gram-negative bacteria harbor multiple cephalosporin and carbapenem resistance genes, highlighting widespread antimicrobial resistance (Sands et al., 2021).

Local data is crucial for informing treatment strategies due to regional variations in bacterial spectrum and antimicrobial sensitivity patterns (Poyekar, 2022; Moni et al., 2020; Uwe et al., 2022; Verma et al., 2015). The burden of neonatal sepsis is worsened by the scarcity of accurate information on its causes and consequences in developing countries, including Ethiopia. Additionally, most studies are limited by small sample sizes (Sands et al., 2021).

Therefore, this study aimed to address the data gap by determining the most common bacterial etiologies of neonatal sepsis in Gondar Comprehensive Specialized Hospital (UoGCSH), a critical healthcare hub in northwestern Ethiopia, and assessing the antibiotic resistance patterns of these key pathogens. The findings will inform targeted treatment strategies to improve outcomes for critically ill neonates in the local hospital’s care and contribute valuable regional data to national efforts to combat the public health challenge of neonatal sepsis.

## Materials and methods

### Study design and setting

A cross-sectional study was conducted on neonates (aged ≤28 days) suspected of bloodstream infections who were admitted to the UoGCSH from January 2019 to December 2021. The hospital is located in the center of Gondar town, approximately 747 kilometers northwest of Addis Ababa, the capital city of Ethiopia. The hospital is a leading healthcare institution in the region and serves as a referral center for over 7 million people, catering to a diverse population from both urban and rural areas (Gobezie et al., 2023). The hospital is equipped with specialized facilities and a dedicated neonatal unit, which is essential for managing cases of neonatal sepsis. It offers various specialized diagnostic services to neonates and children. The neonatal unit is equipped with advanced medical facilities and staffed by skilled healthcare professionals, including pediatricians and nurses, who are dedicated to managing complex cases of neonatal sepsis. The hospital has several laboratory departments, including medical microbiology, clinical chemistry, hematology, serology, medical parasitology, and one main laboratory room. The microbiology laboratory plays a vital role in enabling the collection and analysis of blood samples to identify bacterial pathogens and assess their antibiotic susceptibility profiles.

### Blood culture and bacterial identification

Standardized protocol-guided blood collection and bacterial identification were implemented. Two milliliters of blood were aseptically collected from each neonate and inoculated at a 1:10 ratio (blood: broth) into sterile Tryptone Soy Broth. Culture bottles incubated at 35-37°C for up to 7 days with daily monitoring for growth signs (hemolysis, turbidity, clot formation). Positive cultures underwent Gram staining and subculture onto various selective and differential media (blood agar, chocolate agar with 5% CO2, MacConkey agar, and mannitol salt agar) for further differentiation. These plates were incubated aerobically at 37°C for 18–24 h.

A two-step approach identified bacterial isolates. Initially, colony characteristics (color, size, shape, texture) were examined macroscopically. Subsequently, Gram-negative isolates underwent various conventional biochemical tests (indole, urease, lysine decarboxylase, triple sugar iron agar, citrate utilization, oxidase, and motility tests) for further differentiation. Gram-positive identification relied on Gram staining, catalase activity, coagulase testing, and hemolytic pattern analysis. This combined approach ensured comprehensive and reliable bacterial pathogen identification (Arega et al., 2018; Arega et al., 2017).

### Antimicrobial susceptibility testing

The Kirby-Bauer disk diffusion method determined antimicrobial susceptibility patterns. Briefly, a standardized suspension of bacterial isolates was prepared in saline and adjusted to a 0.5 McFarland standard. This suspension was then inoculated onto Mueller-Hinton agar (non-fastidious bacteria) or Mueller-Hinton agar supplemented with 5% sheep blood (fastidious bacteria). Following inoculation, commercially available antibiotic disks (erythromycin, clindamycin, ampicillin, etc.) were applied, and plates were incubated at 37°C for 18–24 h. The diameters of inhibition zones surrounding each disk were measured, and susceptibility was categorized as sensitive, intermediate, or resistant according to the 2019 CLSI guidelines (Clinical and Laboratory Standards Institute, 2019).

### Data extraction

The primary data source for this study was the records from the microbiology laboratory at UoGCSH. Six experienced laboratory professionals were involved in the data collection process guided by a standardized checklist. This checklist captured demographic information (age, gender), clinical setting (ICU admission), admission date, presenting complaints (fever, hypothermia), prior antibiotic use, culture and identification results, and susceptibility testing results for a broad spectrum of antibiotics. Antibiotic susceptibility results of intermediate susceptibility were categorized as “resistant” for analysis purposes.

### Operational and case definitions

#### Antimicrobial susceptibility pattern

The response of specific bacterial isolates to various antibiotics, categorized as resistant, intermediate, or susceptible based on inhibition zone diameters. We categorized both “resistant” and “intermediate” patterns as resistant.

#### Multidrug resistance

The ability of a bacterial strain to resist three or more antimicrobial agents from different classes (Alam et al., 2011).

### Data management and analysis

Data were checked for completeness and encoded in an Excel spreadsheet. Then, the data were exported to STATA version 17 for analysis. Descriptive statistics (frequency and percentage) were computed. Pearson’s chi-square test was used to assess the association between neonatal sepsis and potential risk factors. A *p*-value of less than 0.05 was considered statistically significant. Finally, the study results are presented in text, tables, and figures as appropriate.

### Sample and data quality control

Standard operating procedures for microbiological techniques were followed throughout blood sample collection, transportation, culture media inoculation and incubation, and biochemical testing. Culture media sterility was ensured by random selection and incubation of 5% of prepared media. Media performance was regularly evaluated using known standard strains of *E. coli* (ATCC 25922), *S. aureus* (ATCC 25923), and *P. aeruginosa* (ATCC 27853). Microbiology experts monitored culture media inoculation, colony characterization, measurement, and interpretation of antibiotic susceptibility tests. The investigators developed a standardized data extraction form, and its accuracy, completeness, consistency, and reliability were assessed using a pilot study involving a random sample of 100 patient records.

### Ethical consideration

Before commencing the research, the authors ensured adherence to ethical guidelines. They obtained ethical approval from the University of Gondar Institutional Review Board (IRB). Additionally, a letter of support from the College of Medicine and Health Sciences facilitated data collection. To ensure participant anonymity, patient personal information was omitted, and data were analyzed anonymously. Since the study was retrospective, the IRB waived the requirement for informed consent as obtaining consent from past participants would be impractical. Furthermore, to strengthen confidentiality, no personal identifiers were used, and only the investigator had access to the collected data. The research was conducted following the Declaration of Helsinki.

## Results

### Socio-demographic and clinical characteristics of study participants

A total of 1,236 study participants were enrolled in the study. The majority, 747 (60.4%), were male, and 772 (62.4%) were aged less than 7 days. The primary clinical setting was the NICU, which accounted for 1,215 (98.2%) of the participants. The distribution of cases by year of diagnosis showed that the highest number of cases, 680 (55.0%), occurred in 2021. A significant proportion of patients, 1,174 (95.2%), experienced fever, with 1,190 (96.2%) reporting fever onset before admission. Furthermore, 384 (31.0%) had received antibiotic therapy within 2 days before admission. Increased respiration was observed in 503 (40.7%) participants, and a similar proportion, 503 (40.7%), were suspected of having pneumonia (Table 1).

**Table 1:** Socio-demographic characteristics of bloodstream infections suspected study participants at UoGCSH, Ethiopia (*n* = 1,236).

Variables	Category	Frequency	Percentage (%)
Gender	Male	747	60.4
	Female	490	39.6
Age in days	≥7	465	37.6
	<7	772	62.4
Clinical setting	NICU	1,215	98.2
	Preterm	22	1.8
Year of diagnosis	2019	252	20.4
	2020	304	24.6
	2021	680	55.0
Fever	Yes	1,174	95.2
	No	59	4.8
Fever before admission	Yes	1,190	96.2
	No	47	3.8
Hypothermia	Yes	29	2.3
	No	1,208	97.7
Hypotension	Yes	25	2.0
	No	1,212	98.0
Increased respiration	Yes	503	40.7
	No	734	59.3
Suspicion of pneumonia	Yes	503	40.7
	No	734	59.3
Suspicion meningitis	Yes	164	13.3
	No	1,073	86.7
Suspicion neonatal infection	Yes	205	16.6
	No	1,031	83.4
Antibiotic therapy within 2 days before admission	Yes	384	31.0
	No	853	69.0
Sign of microbial growth within 2 days of incubation	Yes	403	32.7
	No	829	67.3

### Prevalence of bacterial isolates

**Figure 1:**
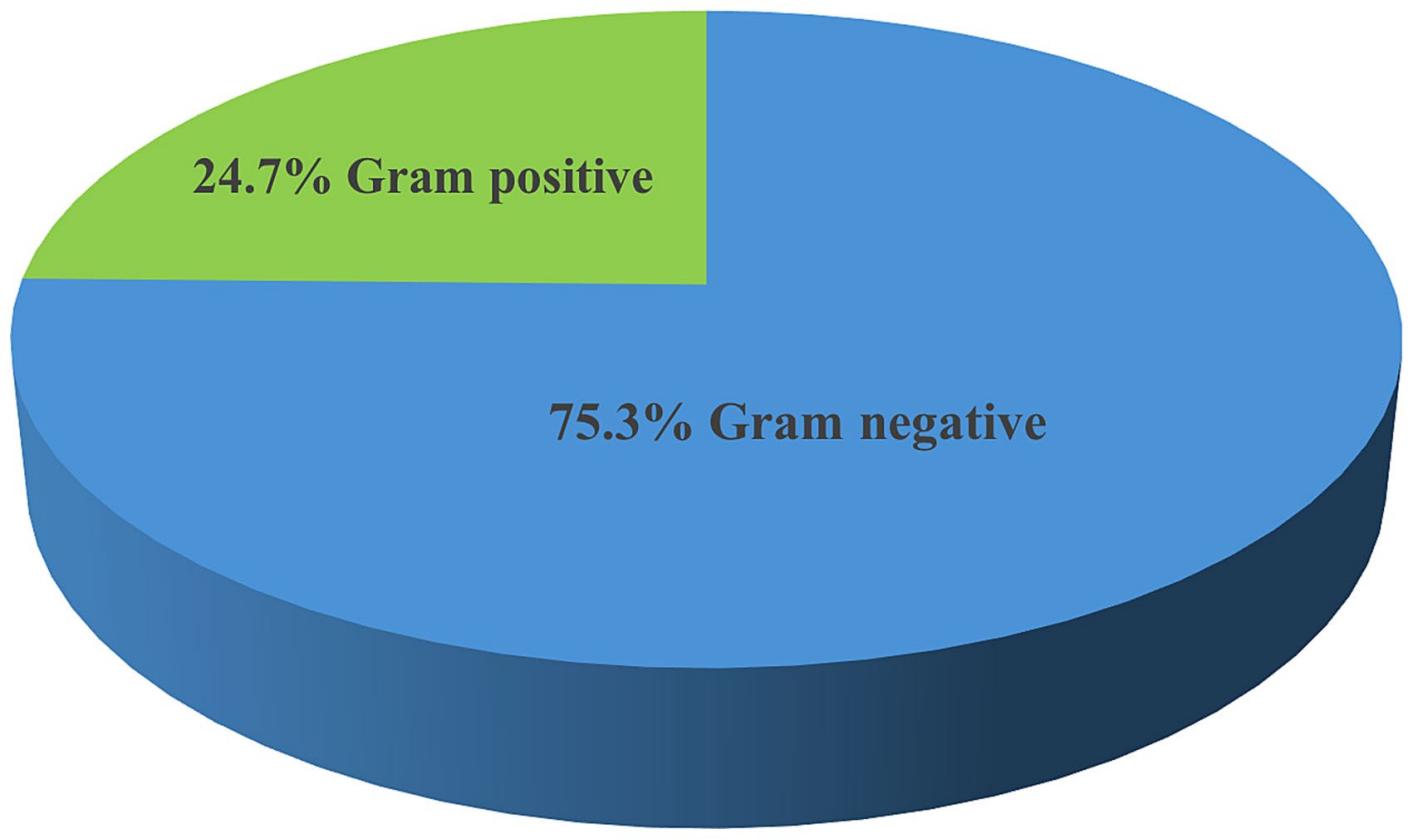
Frequency of bacterial pathogens isolated from neonates suspected of bloodstream infections at UoGCSH, 2024.

The overall prevalence of microbial isolates was 25.4% (314/1236). Among this, bacterial pathogens and yeast cells accounted for 90.5% (284/314) and 9.6% (30/314), respectively. The prevalence of bacterial pathogens from the overall septicemia suspected patients was 23% (284/1236). Among the bacterial isolates, Gram-negative bacteria were predominant, comprising 75.3% (214/284) of the identified pathogens, while Gram-positive bacteria accounted for 24.7% (70/284). Additionally, 4 *Bacillus* spp. isolates were identified as contaminants (Figure 1). The most frequently isolated bacterial pathogens were *K. pneumoniae* at 38.7% (110/284), followed by *S. aureus* at 13% (37/284), and *Acinetobacter* spp. at 8.1% (30/284). Other significant findings included *E. coli* at 6.3% (18/284), and Non-fermenting Gram-negative rods (NFGNR) at 6% (17/284) (Table 2).

**Table 2:** Frequency of bacterial pathogens isolated from patients suspected of septicemia at the UoGCSH, Ethiopia, 2024 (*n* = 284 isolates).

Bacterial isolates	Frequency	Percentage (%)
*K. pneumoniae*	110	38.7
*S. aureus*	37	13.0
*Acinetobacter* spp.	23	8.1
*E. coli*	18	6.3
*NFGNR*	17	6.0
*E. cloacae*	15	5.3
*S. viridans*	15	5.3
*K. ozaenae*	9	3.2
*K. oxytocia*	8	2.8
*Enterococcus* spp.	7	2.5
*Citrobacter* spp.	3	1.1
*P. aerogensa*	3	1.1
*S. typhi*	3	1.1
*S. agalactiae*	3	1.1
Others*	13	4.6
Total	284	100.0

Others* (CoNS, *P. mirabilis*, *P. stuartii*, *S. pyogens*, *K. rhinoscleromatis*, *N. meningitides*, *Salmonella Group* A, *Serratia* spp., *S. pneumoniae*).

### Antimicrobial susceptibility patterns of bacterial isolates

Among Gram-positive isolates, CoNS exhibited 100% (2/2) resistance to penicillin, oxacillin, and gentamicin. *S. aureus* showed a high resistance rate of 94.6% (35/37) of isolates to penicillin, 64.9% to oxacillin, and 40.5% to gentamicin. Similarly, for *S. viridans* 73.3% (11/15) of isolates were resistant to ampicillin and 20% (3/15) were resistant to vancomycin. The single isolate of *S. pneumoniae* showed no resistance to the tested antibiotics. Enterococcus species demonstrated 85.7% (6/7) resistance to penicillin, 57% (4/7) to vancomycin, and 71.4% (5/7) to chloramphenicol. *S. agalactiae* exhibited no resistance to ampicillin and vancomycin antimicrobial agents. *S. pyogenes* showed 50% (1/2) resistance to vancomycin. This study highlights significant resistance patterns, especially in CoNS, *S. aureus*, and *Enterococcus* spp., necessitating careful consideration of antibiotic selection for effective treatment (Table 3).

**Table 3:** Antimicrobial sensitivity test result for Gram-positive bacterial pathogens isolated from suspected bloodstream infections at UoGCSH, Ethiopia, 2024.

Isolated organisms (*n*)	Anti-microbial susceptibility test						
	PEN	AMP	OXC	CIP	GEN	VAN	CAF
	R *n* (%)	R *n* (%)	R *n* (%)	R *n* (%)	R *n* (%)	R *n* (%)	R *n* (%)
CoNS (2)	2 (100)	Na	2 (100)	Na	2 (100)	Na	Na
*S. aureus* (37)	35 (94.6)	Na	24 (64.9)	Na	15 (40.5)	Na	Na
*S. viridans* (15)	Na	11 (73.3)	Na	Na	Na	3 (20)	Na
*S. pneumoniae* (1)	Na	0	Na	Na	Na	0	Na
*Enterococcus* spp. (7)	6 (85.7)	Na	Na	Na	Na	4 (57)	5 (71.4)
*S. agalactiae* (3)	Na	0	Na	Na	Na	0	Na
*S. pyogenes* (2)	Na	0	Na	Na	Na	1 (50)	Na

PEN, Penicillin; AMP, Ampicillin; OXC, Oxacillin; CIP, Ciprofloxacin; GEN, Gentamicin; VAN, Vancomycin; CAF, Chloramphenicol; Na, Not applicable.

A significant burden of antimicrobial resistance among Gram-negative bacterial isolates recovered among the study participants. Among the isolated bacterial pathogens, *K. pneumoniae* was the most concerning resistance profile for meropenem (88.1%), ceftazidime (83.6%), ceftriaxone (83.6%), and amoxicillin-clavulanate (69%). Conversely, *E. coli* showed relatively lower resistance rates, with the highest resistance observed for ampicillin (66.7%), gentamicin (61.1%), and amoxicillin-clavulanate (55.6%). *Acinetobacter* spp. exhibited moderate resistance to ceftriaxone (52.2%), ceftazidime (47.8%), and amoxicillin-clavulanate (47.8%), but remained largely susceptible to meropenem (13%) and ciprofloxacin (21.7%). *E. cloacae* isolate showed high resistance to ampicillin (93.3%) and amoxicillin-clavulanate (86.7%), with moderate resistance to other antibiotics tested. The NFGNR group displayed variable resistance patterns, with the highest rates observed for ceftazidime (64.7%) and ceftriaxone (64.7%). Among bacterial isolates, *Bacillus* spp. were more likely contaminants and has not been tested for antimicrobial agents. Furthermore, *K. ozaenae* isolates exhibited particularly high resistance to ceftazidime (88.9%), ceftriaxone (88.9%), and trimethoprim-sulfamethoxazole (88.9%) (Table 4).

**Table 4:** Antimicrobial sensitivity test results for Gram-negative bacterial isolates from bloodstream infections suspected neonates at UoGCSH, Ethiopia.

Isolated organisms (*n*)	Anti-microbial susceptibility test												
	AMP	AMC	PTZ	CAZ	CRO	CIP	MER	SXT	GEN	AMK	CAF	TOB	PEF
	R *n* (%)	R *n* (%)	R *n* (%)	R *n* (%)	R *n* (%)	R *n* (%)	R *n* (%)	R *n* (%)	R *n* (%)	R *n* (%)	R *n* (%)	R *n* (%)	R *n* (%)
*K. pneumoniae* (110)	Na	76 (69)	67 (61)	92 (83.6)	92 (83.6)	79 (71.8)	54 (49)	96 (88.1)	83 (75.4)	71 (64.5)	81 (73.7)	83 (75.5)	78 (70.9)
*E. coli* (18)	12 (66.7)	10 (55.6)	9 (50)	8 (44.4)	8 (44.4)	10 (55.6)	5 (27.8)	9 (50)	11 (61.1)	9 (50)	10 (55.6)	11 (61.4)	8 (44.4)
*Acinetobacter* spp. (23)	Na	Na	11 (47.8)	11 (47.8)	12 (52.2)	5 (21.7)	3 (13)	Na	8 (34.8)	8 (34.8)	Na	8 (34.8)	6 (26)
*E. cloacae* (15)	14 (93.3)	13 (86.7)	9 (60)	9 (60)	8 (53.3)	5 (33.3)	6 (40)	6 (40)	6 (40)	8 (53.3)	6 (40)	6 (40)	5 (33.3)
*NFGNR* (17)	Na	Na	6 (35.3)	11 (64.7)	11 (64.7)	5 (29.4)	6 (35.3)	Na	5 (29.4)	4 (23.5)	Na	4 (23.5)	3 (17.6)
*K. ozaenae* (9)	Na	7 (77.8)	4 (44.4)	8 (88.9)	8 (88.9)	5 (55.6)	6 (66.7)	8 (88.9)	6 (70)	4 (40)	7 (77.8)	7 (70)	6 (60)
*P. aeruginosa* (3)	Na	Na	2 (66.7)	3 (100)	2 (66.7)	0	0	Na	1 (33.3)	1 (33.3)	1 (33.3)	1 (33.3)	0
*Citrobacter* spp. (3)	Na	Na	0	1 (33.3)	1 (33.3)	0	0	1 (30)	1 (33.3)	0	1 (30)	1 (33.3)	0
*P. stuartii* (2)	1 (50)	0	0	1 (50)	1 (50)	0	0	0	0	0	0	0	0
*K. oxytoca* (8)	Na	7 (87.5)	7 (87.5)	8 (100)	8 (100)	7 (87.5)	6 (75)	8 (100)	6 (75)	4 (50)	4 (50)	7 (87.5)	7 (87.5)
*P. mirabilis* (2)	2 (100)	0	0	0	0	0	2 (100)	2 (100)	0	2 (100)	0	0	0
*K. rhinoscleromatis* (1)	Na	0	0	0	0	0	0	0	0	0	0	0	0
*S. typhi* (3)	3 (100)	2 (66.7)	1 (33.3)	2 (66.7)	2 (66.7)	0	1 (33.3)	2 (66.7)	2 (66.7)	0	0	2 (66.7)	0
*Salmonella* group A (1)	1 (100)	1 (100)	0	1 (100)	1 (100)	0	1 (100)	1 (100)	1 (100)	0	0	1 (100)	0
*Serratia* spp. (1)	0	0	0	0	0	0	0	0	1 (100)	0	0	0	0
*N. meningitides* (1)	Na	Na	Na	Na	0	0	0	0	Na	Na	Na	Na	0

AMP, Ampicillin; AMC, Amoxicillin-clavulanate; PTZ, Piperacillin_tazobactam; CAZ, Ceftazidime; CRO, Ceftriaxone; CIP, Ciprofloxacin; MER, Meropenem; SXT, Trimethoprim-sulfamethoxazole; GEN, Gentamycin; AMK, Amikacin; CAF, Chloramphenicol; TOB, Tobramycine; PEF, Pefloxacin; Na, Not applicable.

### Multidrug resistance pattern of bacterial isolates

The isolates exhibited a concerning level of multidrug resistance with 61.6% (175/284) of the isolates being resistant to three or more antibiotic classes. The data shows that *K. pneumoniae* had the highest rate of multidrug resistance, with 90.9% (100/284) of the isolates being resistant to three or more antibiotic classes. Notably, *K. ozaenae, K. oxytocia,* and *P. mirabilis* exhibited 100% (2/2) multidrug resistance. Other bacteria such as *S. aureus,* Acinetobacter spp., *E. coli,* and NFGNR also demonstrated high MDR rates at 40.5% (15/284), 43.5% (10/284), 61.1% (11/284), and 41.2% (7/284), respectively. On the other hand, Enterococcus spp. and *S. viridans S. agalactiae*, CoNS, *P. stuartii*, and *S. pyogens* did not show any multidrug resistance. The data indicates a concerning level of multidrug resistance among the bacterial isolates, with 61.6% of the total isolates being resistant to three or more antibiotic classes (Table 5).

**Table 5:** Multidrug resistance patterns of bacterial isolates from neonates suspected of bloodstream infections at UoGCSH, 2024.

Bacterial isolates	Degree of microbial resistance					Total MDR isolates ≥ R3 *n* (%)
	R0	R1	R2	R3	R4	
*K. pneumoniae* (110)	3	3	4	3	97	100 (90.9)
*S. aureus* (37)	13	9	0	15	0	15 (40.5)
*Acinetobacter* spp. (23)	7	3	3	1	9	10 (43.5)
*E. coli* (18)	4	1	2	1	10	11 (61.1)
*NFGNR* (17)	3	2	5	1	6	7 (41.2)
*E. cloacae* (15)	2	4	2	0	7	7 (46.7)
*S. viridans* (15)	5	9	1	0	0	0 (0)
*K. ozaenae* (9)	0	0	0	0	9	9 (100)
*Enterococcus* spp. (7)	1	3	3	0	0	0 (0)
*K. oxytocia* (8)	0	0	0	0	8	8 (100)
*Citrobacter* spp. (3)	1	0	1	0	1	1 (33.3)
*P. aeruginosa* (3)	0	1	0	1	1	2 (66.7)
*S. typhi* (3)	1	0	0	0	2	2 (66.7)
*S. agalactiae* (3)	3	0	0	0	0	0 (0)
CoNS (2)	0	0	2	0	0	0 (0)
*P. mirabilis* (2)	0	0	0	2	0	2 (100)
*P. stuartii* (2)	1	0	1	0	0	0 (0)
*S. pyogens* (2)	1	0	1	0	0	0 (0)
*K. rhinoscleromatis* (1)	1	0	0	0	0	0 (0)
*N. meningitides* (1)	1	0	0	0	0	0 (0)
*S. proup* A (1)	0	0	0	0	1	1 (100)
*Serratia* spp. (1)	0	1	0	0	0	0 (0)
*S. pneumoniae* (1)	1	0	0	0	0	0 (0)
Total n (%)	16.9 (48/284)	12.7 (36/284)	8.8 (25/284)			61.6 (175/284)

R0: no antibiotic resistance; R1: resistance to one category of antimicrobial agent; R2: resistance to two different categories of antimicrobial agents; R3: resistance to three different categories of antimicrobial agents; R4: ≥ resistance to four different categories of antimicrobial agents.

### Factors associated with bloodstream infections

This analysis investigated factors associated with bloodstream infections using Pearson’s chi-square test. While no significant association was found between positive blood cultures and gender, fever before admission, fever after admission, and hypothermia, several other factors emerged as important. Patients with increased respiration and suspected pneumonia were more likely to have positive cultures (*p* = 0.004). Likewise, suspected meningitis also showed a significant association (*p* = 0.009). Interestingly, prior antibiotic use did not statistically influence the results. Notably, the year of admission was the only demographic factor with a significant association. Patients admitted in 2021 had a higher proportion of positive cultures compared to those admitted in 2019 or 2020 (*p* < 0.001) (Table 6).

**Table 6:** Factors associated with positive bacterial blood cultures among neonates with suspected bloodstream infection at UoGCSH (2019–2021).

Variables	Category	Blood culture result		Pearson *x*^2^	*p*-value
		Negative *n* (%)	Positive *n* (%)		
Gender	Male	547 (44.2)	200 (16.2)	1.592	0.207
	Female	373 (30.2)	117 (9.5)		
Age in years	>7	573 (46.3)	199 (16.1)	0.161	0.689
	≤7	347 (28.1)	118 (9.5)		
Year of admission	2019	165 (13.4)	87 (7.0)	15.666	<0.001*
	2020	240 (19.4)	64 (5.2)		
	2021	514 (41.6)	166 (13.4)		
Fever	Yes	872 (70.7)	302 (24.5)	0.004	0.948
	No	44 (3.6)	15 (1.2)		
Fever before admission	Yes	885 (71.5)	305 (24.7)	0.000	0.986
	No	35 (2.8)	12 (1.0)		
Hypothermia	Yes	20 (1.6)	9 (0.7)	0.305	0.581
	No	900 (72.8)	308 (24.9)		
Hypotension	Yes	16 (1.3)	9 (0.7)	1.080	0.299
	No	904 (73.1)	308 (24.9)		
Increased respiration	Yes	391 (31.6)	112 (9.1)	8.254	0.004*
	No	529 (42.8)	205 (16.6)		
Suspicion of pneumonia	Yes	391 (31.6)	112 (9.1)	8.254	0.004*
	No	529 (42.8)	205 (16.6)		
Suspicion of meningitis	Yes	137 (11.1)	27 (2.2)	6.868	0.009*
	No	783 (63.3)	290 (23.4)		
Suspicion of neonatal infection	Yes	142 (11.5)	63 (5.1)	0.883	0.348
	No	777 (62.9)	254 (20.6)		
Antibiotic therapy before laboratory diagnosis	Yes	279 (22.6)	105 (8.5)	0.432	0.511
	No	641 (51.8)	212 (17.1)		

*Statistically significant association.

## Discussion

Neonatal sepsis is a significant threat to newborn health, particularly in developing countries like Ethiopia, where it is a leading cause of mortality and morbidity (Weldu et al., 2020; Wen et al., 2021; Klingenberg et al., 2018; Sherif et al., 2023). Accurately identifying bacterial pathogens causing neonatal sepsis and their antibiotic susceptibility patterns is crucial for effective patient management. This information helps guide treatment strategies and improves patient outcomes. Therefore, this study aimed to characterize the bacterial profiles and antibiotic susceptibility at UoGCSH in Ethiopia.

The current study found that nearly a quarter of neonates suspected of sepsis had confirmed bacterial infections (23%). This prevalence is comparable to studies conducted in China (23%) (Fang et al., 2023), Tanzania (24%) (Mhada et al., 2012), Ghana (21.0%) (Acheampong et al., 2022), Nepal (20.5%) (Pokhrel et al., 2018), and Ethiopia (21%) (Sherif et al., 2023). However, the finding is higher than results from Bhutan (14%) (Jatsho et al., 2020), Nepal (10.8%) (Thapa and Sapkota, 2019), Uganda (12.8%) (Tumuhamye et al., 2020), Iran (15.98%) (Akbarian-Rad et al., 2020), South Africa (11.0%) (Reddy et al., 2021), Pakistan (8.9%) (Atif et al., 2021), and Ghana (17.3%) (Aku et al., 2018). Conversely, the current finding is much lower than those reported in various settings in Ethiopia (36.5–46.6%) (Weldu et al., 2020; Worku et al., 2022; Mezgebu et al., 2023; Assemie et al., 2020; Geyesus et al., 2017), Zambia (38%) (Egbe et al., 2023), Nigeria (49.6%) (Peterside et al., 2015), Tanzania (72%) (Majigo et al., 2023), and Uganda (59.0%) (Zamarano et al., 2021). The discrepancy in prevalence rates across different geographical regions could be attributed to factors such as differences in hygiene practices, antibiotic use patterns, variations in study design, and broader epidemiological factors. Additionally, improvements in diagnostic techniques, changes in hospital practices, and seasonal trends could potentially influence the incidence and prevalence of neonatal sepsis.

Among the neonates suspected of septicemia, only 25.4% tested positive for cultures. Several factors could explain the relatively low rate of culture positivity. These include the use of antibiotic treatment before admission, which could suppress bacterial growth, the limitations of conventional culture methods, and the possibility of non-bacterial infections presenting with similar clinical signs and symptoms. Nevertheless, in cases where classical sepsis symptoms were present, but no microbial isolates were obtained, management primarily involved empirical antibiotic therapy based on clinical judgment and local guidelines.

The study revealed that Gram-negative bacteria were predominant, accounting for 75.3% of the isolates, compared to Gram-positive bacteria. This result aligns with previous research from Ethiopia, South Africa, Germany, Tanzania, Uganda, and Nepal, where Gram-negative bacteria were also the majority (Pokhrel et al., 2018; Tessema et al., 2021; Sherif et al., 2023; Majigo et al., 2023; Zamarano et al., 2021; Thomas et al., 2024). However, this percentage is significantly higher than the 58.1% Gram-negative bacteria predominance reported in a systematic review from Iran (Moftian et al., 2023). Among the bacterial pathogens isolated, *K. pneumoniae* was the most frequently isolated followed by *S. aureus* and *Acinetobacter* spp. This finding is supported by a systematic review and a review in Sub-Saharan Africa, Uganda, and Pakistan (Tumuhamye et al., 2020; Atif et al., 2021; Okomo et al., 2019). The high frequency of *K. pneumoniae* isolates in the current study is particularly concerning, as this pathogen is known to be a common cause of healthcare-associated infections and is often associated with multidrug resistance. The prevalence of *S. aureus* and *Acinetobacter* spp. is also significant, as these bacteria can be challenging to treat due to their ability to develop resistance to various antimicrobial agents.

The antimicrobial susceptibility data revealed alarming levels of resistance among both gram-positive and gram-negative bacterial isolates. This concerning trend underscores the critical need for alternative therapeutic strategies and judicious antibiotic use to combat the growing challenge of antimicrobial resistance. Among the gram-positive bacteria, CoNS exhibited 100% resistance to penicillin, oxacillin, and gentamicin, indicating that these common antimicrobial agents are no longer effective against CoNS infections. Similarly, *S. aureus* showed high resistance rates to penicillin (94.6%), oxacillin (64.9%), and gentamicin (40.5%), suggesting that empiric treatment with these drugs may be increasingly ineffective. *S. viridans* also demonstrated significant resistance to ampicillin (73.3%) and vancomycin (20%), which are commonly used therapeutic options for streptococcal infections. These findings highlight the urgent need for new antimicrobial agents and strategies to address the rising threat of antimicrobial resistance.

Gram-negative bacterial isolates also exhibited substantial resistance patterns. *K. pneumoniae* showed the most alarming resistance to highly active antimicrobial agents, including meropenem (88.1%), ceftazidime (83.6%), ceftriaxone (83.6%), and amoxicillin-clavulanate (69%). This indicates that clinicians may have limited treatment options for *K. pneumoniae* infections, as these drugs are often considered last-line or “reserve” antimicrobials. Although *E. coli* showed relatively lower resistance rates compared to *K. pneumoniae*, it still presented significant resistance to ampicillin (66.7%), gentamicin (61.1%), and amoxicillin-clavulanate (55.6%). These findings are consistent with the previous study from the Tigray region, Ethiopia, in which *K. pneumoniae* and *E. coli* were resistant to common antimicrobial agents (Weldu et al., 2020). These observed patterns of resistance to commonly used antimicrobial drugs, such as ampicillin, ceftazidime, ceftriaxone, gentamicin, and amoxicillin-clavulanic acid, have been reported in other studies as well (Bai et al., 2021; Weldu et al., 2020; Sherif et al., 2023), suggesting that these resistance trends are widespread and not limited to the specific setting of this study.

Furthermore, the majority (61.6%) of the bacterial isolates were MDR, although this rate is lower than those reported in some other studies where the proportion of MDR was as high as 84% (Sherif et al., 2023; Zenebe et al., 2021). Among specific bacterial pathogens, *K. pneumoniae* had the highest rate of MDR, with 90.9% of the isolates being resistant to three or more antibiotic classes. Other bacterial pathogens, including *K. ozaenae*, *K. oxytoca*, and *P. mirabilis*, also exhibited 100% MDR. Additionally, *S. aureus*, Acinetobacter spp., *E. coli*, and non-fermenting Gram-negative rods demonstrated high MDR rates of 40.5, 43.5, 61.1, and 41.2%, respectively. The high levels of MDR observed in this study are concerning and call for stringent antibiotic stewardship programs to mitigate the spread of resistant strains. Multidrug resistance is particularly alarming as it severely limits the available treatment options and increases the risk of treatment failures. These resistance patterns highlight the urgent need for continuous surveillance and the development of new antimicrobial agents to effectively address the growing threat of antimicrobial resistance.

The study identified several key factors significantly associated with neonatal sepsis. One notable finding was the correlation between the year of admission and the incidence of neonatal sepsis. This temporal trend could be influenced by various factors, including changes in hospital practices, hygiene protocols, and seasonal trends. Additionally, variations in antibiotic resistance patterns, broader epidemiological factors, and improvements in diagnostic techniques could also contribute to these yearly fluctuations. Another important risk factor identified was rapid breathing, which can serve as an early clinical sign of sepsis, often indicating an underlying infection that requires immediate medical attention. The study found that neonates presenting with increased respiration were more likely to develop sepsis. Furthermore, the association between suspected pneumonia and meningitis with neonatal sepsis highlights the potential for overlapping clinical presentations. These symptoms had a higher likelihood of developing into sepsis, underscoring the importance of vigilant clinical assessment and timely intervention in neonatal care.

## Limitations

This study has several important limitations that should be considered when interpreting the results. Since the research was conducted at a single healthcare facility, it may limit the generalizability of the findings to other geographical regions or healthcare settings. The patient population and antimicrobial resistance patterns observed at this one site may not be representative of broader regional or national trends. Furthermore, the study did not include molecular typing of bacterial isolates, which could have provided more detailed insights into the genetic mechanisms underlying antimicrobial resistance and the epidemiology of the infections. The absence of this molecular data limits the depth of understanding regarding the specific strains and resistance patterns present in the bacterial population studied.

## Conclusion

Newborn sepsis caused by highly resistant bacteria presents a significant challenge at the UoGCSH. This study identified a high prevalence of culture-confirmed sepsis, with Gram-negative bacteria, especially *K. pneumoniae*, dominating the isolated pathogens. These bacteria exhibited alarming resistance to commonly used antibiotics, with a very high proportion demonstrating multidrug resistance*. K. pneumoniae* displayed the most concerning resistance rates. Additionally, the study linked specific factors like year of admission, rapid breathing, suspected pneumonia, and suspected meningitis to an increased risk of neonatal sepsis.

## Recommendation

- There should be continuous surveillance of bacterial pathogens causing neonatal sepsis, monitoring their evolving antibiotic susceptibility patterns, which are crucial to inform effective treatment guidelines.
- The hospital should improve diagnostic techniques for the early and accurate identification of bacterial pathogens causing neonatal sepsis.
- The hospital should tailor antibiotic regimens based on the specific bacterial profiles and resistance patterns identified in the local hospital setting to improve the effectiveness of treatments.
- The Federal Ministry of Health and the regional health bureau should develop and implement robust antibiotic stewardship programs to optimize the use of antibiotics, reduce unnecessary prescriptions, and curb the spread of resistant strains.
- The Federal Ministry of Health should develop training programs and provide healthcare providers with the latest guidelines for managing neonatal sepsis.
- Further study should be conducted using molecular techniques to improve the detection of causative pathogens, particularly for those culture-negative patients.
- Researchers should invest significant effort to discover new antimicrobial agents that can effectively combat the resistant strains and provide effective treatment options for neonatal sepsis.

## Data Availability

The original contributions presented in the study are included in the article/supplementary material, further inquiries can be directed to the corresponding author.
